# Mortality of Patients Lost to Follow-Up in Antiretroviral Treatment Programmes in Resource-Limited Settings: Systematic Review and Meta-Analysis

**DOI:** 10.1371/journal.pone.0005790

**Published:** 2009-06-04

**Authors:** Martin W. G. Brinkhof, Mar Pujades-Rodriguez, Matthias Egger

**Affiliations:** 1 Division of International and Environmental Health, Institute of Social and Preventive Medicine (ISPM), University of Bern, Bern, Switzerland; 2 Epicentre, Médecins Sans Frontières, Paris, France; 3 Department of Social Medicine, University of Bristol, Bristol, United Kingdom; University of New South Wales, Australia

## Abstract

**Background:**

The retention of patients in antiretroviral therapy (ART) programmes is an important issue in resource-limited settings. Loss to follow up can be substantial, but it is unclear what the outcomes are in patients who are lost to programmes.

**Methods and Findings:**

We searched the PubMed, EMBASE, Latin American and Caribbean Health Sciences Literature (LILACS), Indian Medlars Centre (IndMed) and African Index Medicus (AIM) databases and the abstracts of three conferences for studies that traced patients lost to follow up to ascertain their vital status. Main outcomes were the proportion of patients traced, the proportion found to be alive and the proportion that had died. Where available, we also examined the reasons why some patients could not be traced, why patients found to be alive did not return to the clinic, and the causes of death. We combined mortality data from several studies using random-effects meta-analysis. Seventeen studies were eligible. All were from sub-Saharan Africa, except one study from India, and none were conducted in children. A total of 6420 patients (range 44 to 1343 patients) were included. Patients were traced using telephone calls, home visits and through social networks. Overall the vital status of 4021 patients could be ascertained (63%, range across studies: 45% to 86%); 1602 patients had died. The combined mortality was 40% (95% confidence interval 33%–48%), with substantial heterogeneity between studies (P<0.0001). Mortality in African programmes ranged from 12% to 87% of patients lost to follow-up. Mortality was inversely associated with the rate of loss to follow up in the programme: it declined from around 60% to 20% as the percentage of patients lost to the programme increased from 5% to 50%. Among patients not found, telephone numbers and addresses were frequently incorrect or missing. Common reasons for not returning to the clinic were transfer to another programme, financial problems and improving or deteriorating health. Causes of death were available for 47 deaths: 29 (62%) died of an AIDS defining illness.

**Conclusions:**

In ART programmes in resource-limited settings a substantial minority of adults lost to follow up cannot be traced, and among those traced 20% to 60% had died. Our findings have implications both for patient care and the monitoring and evaluation of programmes.

## Introduction

In industrialized countries the prognosis of HIV infection has improved considerably since highly active antiretroviral therapy (ART) was introduced from 1995 onwards [1]–[3]. In low-income countries with a high burden of HIV and AIDS, ART has become more widely available in recent years. The World Health Organisation (WHO) estimates that about 3 million people were receiving ART in low- and middle-income countries by the end of 2007, a 7.5-fold increase during the past four years [4].

ART of individual patients and the monitoring and evaluation of treatment programmes critically depend on regular patient follow-up. Individual treatment decisions can then be made and treatment response, complication and mortality rates can be accurately estimated at the programme level [5], [6]. Using data from a network of ART treatment programmes in resource-limited settings, we found that on average 21% of patients had been lost to programmes in the first six months after starting ART [7]. Similarly, a systematic review of ART programmes in sub-Saharan Africa found that about 40% of patients were lost at two years, with large variation in retention rates between programmes [8].

The outcome of patients lost to follow has received relatively little attention. Patients not returning to the clinic where they initiated ART may have stopped taking antiretroviral drugs, resulting in high mortality. Alternatively, with increasing availability of ART, patients may have transferred to another programme, for example a programme closer to their place of residence. We performed a systematic review and meta-analysis of studies that determined the vital status of patients who were lost to follow-up (LTFU) after starting ART in low or middle-income countries. Our objectives were to describe mortality and causes of death among patients LTFU, to examine the reasons why patients LTFU could not be traced and why those traced alive had not returned to the clinic. Our aims were to inform the adjustment of mortality estimates for LTFU, to identify critical issues in patient registration and follow-up and inform strategies to improve patient retention and ascertainment of outcomes.

## Methods

### Data sources

We aimed to identify studies that determined the vital status of all or a subset of patients lost to follow-up after starting ART in treatment programmes in Africa, Asia or Latin America. We searched the PubMed, EMBASE, Latin American and Caribbean Health Sciences Literature (LILACS), Indian Medlars Centre (IndMed) and African Index Medicus (AIM) databases. We limited the search to studies in humans; studies from Africa, Asia or Latin America; and studies published between January 1, 2000 and January 9, 2009. In PubMed we used a combination of free text and Medical Subject Headings (MeSH) and then adapted the search to the other databases. The searches of LILACS and AIM included Spanish, Portuguese and French terms. Further details are given in the Appendix S1.

Using similar keywords we searched the abstract databases of the Conference on Retroviruses and Opportunistic Infections (CROI, 1997–2008) [9]; the Conference on HIV Pathogenesis and Treatment of the International AIDS Society (IAS, 2001–2008) and the International AIDS Conference (AIDS; 2001–2008) [10]. We used Google Scholar [11] to identify electronic publications ahead of print of eligible studies presented at CROI, IAS or AIDS. Finally, we included a study that was accepted for presentation at CROI 2009 conference and co-authored by one of us (M.P-R.).

### Study selection

We included all articles reporting studies where patients LTFU in ART programmes in Africa, Asia or Latin America were actively traced to establish their vital status. We excluded studies from high-income countries, case reports, and studies of patients who were LTFU while not on ART. Two reviewers (M.B. and M.P-R.) independently assessed the eligibility of articles and abstracts. Discrepancies were resolved in consultation with a third reviewer (M.E.).

### Data extraction

Data were extracted in duplicate by the same two reviewers using a standardised questionnaire that covered the characteristics of the ART programme, including location and country; the number of patients enrolled and on ART; the setting (urban, semi-urban or rural); and whether the programme was public or private. We also extracted the definition of LTFU used in the different studies; the total number of patients LTFU and the number of patients traced; the methods used to trace patients (letter, telephone call, and/or home visits); and whether the study involved both patients on ART and not on ART. Discrepancies were resolved by consensus.

The main outcomes were the number of patients who could not be traced, the number who were found to be alive and the number who had died. For patients LTFU who could not be traced, we examined data on possible reasons. For patients found to be alive we extracted the reasons reported for not returning to the clinic. We classified reasons as transfer to another clinic; stopping treatment because of improved health; hospitalised or being too sick to come to the clinic; stigma and social problems; adverse effects of drugs; logistic problems and economic reasons (including cost for transport) and other reasons. Finally, for patients who were known to have died, we extracted information on the likely cause of death. Causes of death were classified as AIDS defining illness; condition not related to AIDS; unnatural cause; and unknown.

### Statistical analysis

We expressed results as percentages and calculated exact binomial 95% confidence intervals for these percentages. We combined data from several studies using random-effects meta-analysis on the logit scale, and transformed combined estimates back to percentages. We then investigated, for the programmes from sub-Saharan Africa, associations between study characteristics and mortality in patients LTFU using random effects meta-regression. Study characteristics considered were: setting (2 categories: urban vs. rural/urban-rural); definition of LTFU (3 categories: missed 1 or 2 scheduled visits, missed last scheduled visit by 2–6 weeks; missed last scheduled visit by >3 months); method of tracing (3 categories: telephone call, home visit, telephone call and home visit); percentage of patients LTFU included in the survey; and percentage of patients traced and actually retrieved during the survey. Data were analysed using STATA version 10.1 (StataCorp, Texas, USA).

## Results

### Selected studies

**Figure 1:**
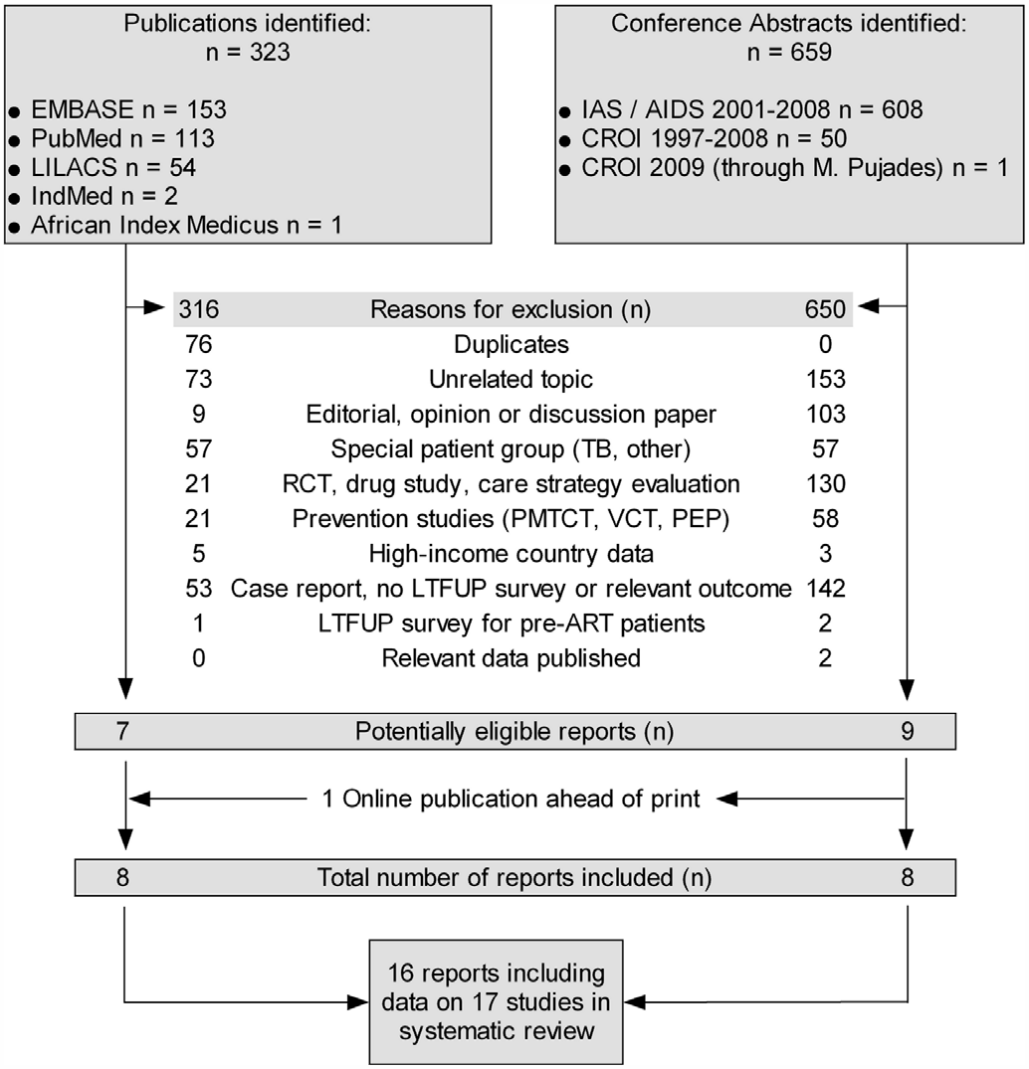
Identification and selection of eligible studies.

Figure 1 describes the process of identifying eligible studies. Among the 323 published items and 659 conference abstracts retrieved, we identified 16 eligible reports (six published articles, one published research letter, one article published electronically ahead of print and eight abstracts), which included data on 17 separate studies.

**Figure 2:**
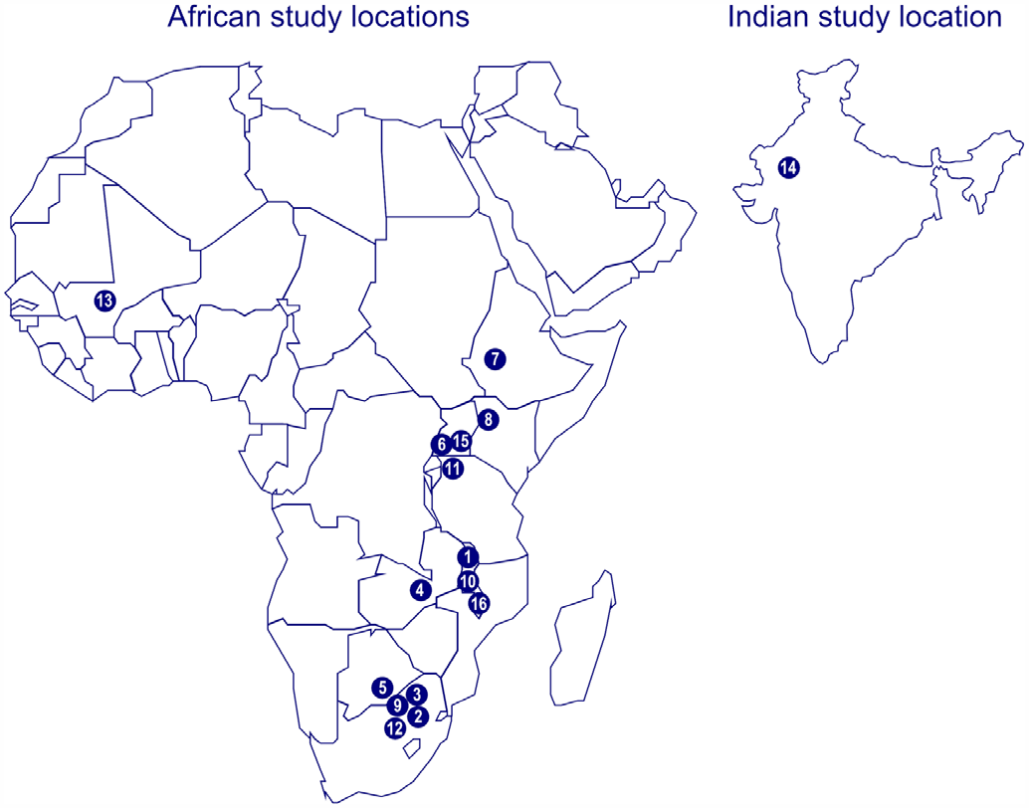
Map of study locations. The numbers refer to Table 1.

Table 1 summarises the characteristics of the 17 studies. One article [12] reported two studies, one from a public ART programme (No. 12 in Table 1) and one from a workplace programme (No. 13) in South Africa. Sixteen were performed in nine sub-Saharan African countries: South Africa (5 studies), Malawi (3 studies), Uganda (2 studies), Zambia, Botswana, Ethiopia, Kenya, Tanzania, and Mali (one study each). One study was from India. Figure 2 shows the geographical location of studies. Two studies (Nos. 4 and 8) [13], [14] included both patients on ART and not on ART. We did not identify any studies in children. Most settings were urban or semi-urban; five studies were from a rural setting. Definitions of LTFU varied. Missing appointments for more than 1 month or more than 3 months was used in several studies (Table 1). Patients were traced using telephone calls, home visits or through social networks. The median duration of follow up from start of ART to last contact in patients LTFU was 1.5 months [15], 2.7 months [16], 4.3 months [17], 13.9 months [18], and not reported in the remaining 12 studies. Median times from start of ART to death in patients LTFU ranged from 1.5 to 2.9 months in the four studies [16], [17], [19], [20] that reported this information. In addition, it is clear that in a further two studies [14], [18] deaths among patients LTFU occurred predominantly in the first 6 months after start of ART.

**Table 1:** Characteristics of ART programmes tracing patients LTFU in low- and middle-income countries.

No.	Study	Location	Setting	LTFU definition	Contact method	Study period	No patients on ART	% LTFU
**Articles**								
1	Yu 2007 [17]	Four facilities in Malawi	Rural	No visit for >3 months	Home visit	2004–2005	5009	5.0
2	Maskew 2007 [20]	Johannesburg, South Africa	Urban	Missed appointments	Telephone	n.r.	5849	n.r.
3	Dalal 2008 [16]	Johannesburg, South Africa	Urban	Missed appointments >6 weeks	Telephone & home visit	2004–2005	1631	16.4
4	Krebs 2008 [13] #	Lusaka, Zambia	Urban & semi-urban	Missed appointments >1 week or month	Home visit	2005	n.r.	21.0*
5	Bisson 2008 [19]	Gaborone, Botswana	Urban	Missed appointments >30 days	Telephone & home visit	2003	410	16.6
6	Geng 2008 [18]	Mbarara, Uganda	Rural	Missed appointments ≥6 months	Home visit	2004–2007	3628	22.9
7	Deribe 2008 [25]	Jimma, Ethiopia	Urban	Missed ≥2 appointments	Telephone & home visit	2007	1270	28.0
8	An 2008 [14] #	Eldoret, Kenya	Urban & rural	Missed appointments	Telephone & home visit	2005–2007	8977	39.3
**Conference abstracts**								
9	Ive 2005 [15]	Johannesburg, South Africa	Urban	Stopped attending the ARV clinic	Telephone	2004–2005	2400	3.1
10	Hochgesang 2006 [21]	Lilongwe, Malawi	Urban	Missed appointments >2 weeks	Home visit	2005	3840	48.0
11	Billy 2007 [23]	Bukoba, Tanzania	Rural	No visit for >3 months	Home visit	2005–2007	1562	17.5
12	Dahab 2008 [12]	Public programme, Gauteng, South Africa	Urban	Missed appointments >1 month	Telephone & home visit^†^	2007	267	16.5
13	Dahab 2008 [12]	Mine programme, Rustenburg, South Africa	Workplace	Missed appointments >1 month	Telephone & home visit	2007	146	36.3
14	Lurton 2008 [24]	Segu region, Mali	Rural	No visit for >3 months	Telephone, social network & home visit	2008	1568	15.1
15	Joshi 2008 [26]	Jodhpur, India	Urban & Rural	No visit for >3 months	Telephone, social network	n.r.	1191	12.8
16	Muwanga 2008 [27]	Kampala, Uganda	Urban	Missed appointments >3 month	Telephone	2007–2008	6421	12.9
17	McGuire 2009 [22]	Chiradzulu, Malawi	Rural	Missed appointments >1 month	Home visit	2008	11057	11.4

n.r.: not reported.

# studies including patients not on ART.

* estimate from Stringer et al. 2006 [30].

### Vital status of patients

**Figure 3:**
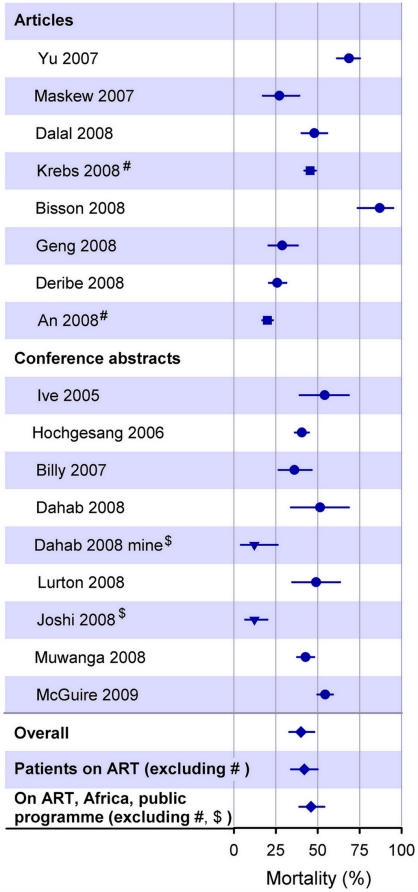
Mortality of patients LTFU that were successfully traced. Study-specific mortality estimates with binomial exact confidence intervals, combined estimates and confidence intervals from random effects meta-analysis. Studies including patients not on ART (^#^ ; squares); workplace programme, programme from outside Africa (^$^ ; triangles).

The number of patients traced and their vital status are summarized in Table 2. Nine studies traced all patients LTFU during the study period and six included a subset of patients representing 15% to 53% of all patients LTFU. Two studies [21], [22] only included patients who had agreed to being traced if LTFU when starting ART. The remaining four studies [14], [18], [23], [24] did not report any criteria for inclusion of patients in the tracing effort. In two other studies [13], [15] the proportion of patients LTFU included in the study was unclear. A total of 6420 patients were traced. Overall, vital status of 4021 patients (63%) could be ascertained; the percentage ascertained ranged from 45% to 87%. A total of 1602 patients had died (40%, range across studies 12% to 87%). The combined mortality from random effects meta-analysis was 40% (95% CI 33%–48%). When removing the two studies that included patients not on ART, mortality increased to 42% (95% CI 34%–50%). When further restricting the analysis to public ART programmes in sub-Saharan Africa (12 studies), mortality was 46% (95% CI: 39%–54%). In all three meta-analyses the between-study heterogeneity was substantial, with I^2^ values >90% and P from tests of heterogeneity <0.0001 (Figure 3).

**Table 2:** Vital status of patients lost to follow up in ART programmes in resource-limited settings.

Study	Number of patients		Vital status of patients lost to follow-up (%)			
	LTFU	Included (%)	Unknown (n)	Alive (n)	Dead (n)	Mortality among traced
**Articles**						
Yu 2007	253	253 (100%)	27% (68)	23% (58)	50% (127)	69%
Maskew 2007	154	154 (100%)	55% (84)	33% (51)	12% (19)	27%
Dalal 2008	267	267 (100%)	35% (94)	34% (90)	31% (83)	48%
Krebs 2008^#^	n.r.	1343 (-)	41% (554)	32% (430)	27% (359)	46%
Bisson 2008	68	68 (100%)	32% (22)	9% (6)	59% (40)	87%
Geng 2008	829	128 (15%)	13% (17)	62% (79)	25% (32)	29%
Deribe 2008	355	355 (100%)	18% (65)	61% (215)	21% (75)	27%
An 2008^#^	3528	1143 (32%)	46% (522)	43% (497)	11% (124)	20%
**Conference abstracts**						
Ive 2005	n.r.	74 (-)	35% (26)	30% (22)	35% (26)	54%
Hochgesang 2006	1843	727 (39%)	26% (189)	44% (320)	30% (218)	41%
Billy 2007	273	113 (41%)	14% (16)	55% (62)	31% (35)	36%
Dahab 2008	44	44 (100%)	20% (9)	39% (17)	41% (18)	51%
Dahab 2008^$^	53	53 (100%)	23% (12)	68% (36)	9% (5)	12%
Lurton 2008	236	61 (26%)	16% (10)	43% (26)	41% (25)	49%
Joshi 2008^$^	152	152 (100%)	30% (46)	61% (93)	9% (13)	12%
Muwanga 2008	831	831 (100%)	55% (459)	26% (213)	19% (159)	43%
McGuire 2009	1233	654 (53%)	32% (206)	31% (204)	37% (244)	54%
Overall		6420 (100%)	37%	38%	25%	40%
Patients on ART (excluding^#^)		3934	34%	38%	28%	42%
On ART, Africa, public programme, (excluding^#, $^)		3729	34%	37%	29%	46%

**Figure 4:**
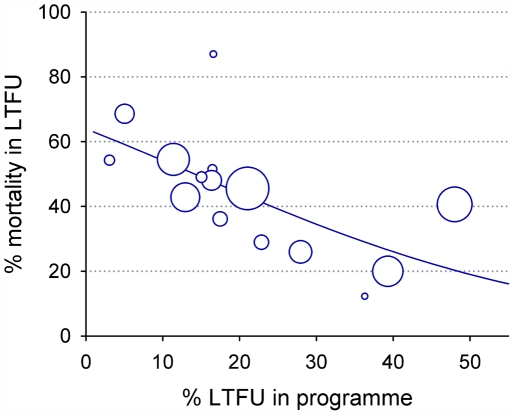
Estimated change in mortality among patients LTFU with proportion of patients LTFU in programme. Analysis based on 15 studies from sub-Saharan Africa. The area of each circle is inversely proportional to the variance of the estimate for that study.

In the studies from sub-Saharan Africa, the percentage of patients LTFU in a programme was associated with mortality in the patients LTFU (p from meta-regression model = 0.02). The estimated mortality in patients LTFU declined from around 60% to 20% as the percentage of patients LTFU in the programme increased from 5% to 50% (Figure 4). The association was similar when excluding the two studies [13], [14] that included some patients not on ART, or the study in a South African mine [12]. There was little evidence that in addition to the percentage of patients LTFU mortality varied with other characteristics, including the setting of the programme (p = 0.51), definition of LTFU used (p = 0.90), method of tracing (p = 0.75), the proportion of patients LTFU included in the survey (p = 0.73), and the proportion of patients successfully traced (p = 0.80).

### Reasons why patients were not found

Seven studies [15]–[17], [20], [23], [25], [26] provided information on the reasons why the tracing of 401 patients was unsuccessful. The majority of patients (333, 83%) were not found because of an incorrect, incomplete or missing telephone number or home address in the patient file. Sixty-four patients (16%) had moved to an unknown location, or a location too far from the clinic to allow a home visit. Reasons were unknown in the remaining patients. Two studies [13], [27] mentioned inadequate contact information as the main reason why tracing failed, but did not give any figures. Three studies compared the characteristics of patients who were found to those who could not be traced [13], [16], [21]. Two of the three studies reported that the patients not found had distributions of clinical stage, CD4 counts and viral load similar to patients found to be alive [13], [16]. However, Hochgesang et al. [21] reported that patients who could not be found had low initial CD4 counts and suggested that many of these might have died.

### Reasons for not returning to the clinic

Reasons for not returning to the clinic among patients found alive were assessed in 11 studies, for 1096 (75%) of the 1464 surviving patients (Table 3). Not all reasons were considered in all programmes, and the importance of different reasons varied across programmes. Common reasons included the transfer to another ART programme, financial problems (for example with costs of transport), and improved or deteriorating health. Stigma and social problems and adverse effects were less frequently mentioned. Other reasons reported in single studies were pregnancy or childbirth [16], advice from care provider [20], administrative problems (for example loss of patient cards) [16] or religious beliefs [13], [17].

**Table 3:** Reasons for not returning to ART programme among patients found alive.

Study	% of patients interviewed (n)	Transfer out	Financial reasons	Improved health	Too sick to come to clinic	Stigma & social problems	Adverse effects of drugs	Other reasons
Yu 2007	100% (58)	35%	22%	n.r.	n.r.	7%	n.r.	36%
Maskew 2007	100% (51)	12%	47%	n.r.	n.r.	8%	2%	31%
Dalal 2008	100% (90)	49%	2%	10%	20%	n.r.	6%	13%
Krebs 2008^#^	63% (271)	n.r.	n.r.	4%	23%	7%	n.r.	67%
Deribe 2008	79% (170)	n.r.	n.r.	n.r.	n.r.	64%	8%	28%
Ive 2005	100% (22)	43%	14%	n.r.	n.r.	n.r.	19%	24%
Billy 2007	97% (60)	35%	n.r.	62%	n.r.	n.r.	n.r.	3%
Lurton 2008	100% (26)	54%	n.r.	n.r.	n.r.	n.r.	n.r.	n.r.
Joshi 2008	92% (86)	14%	45%	3%	n.r.	n.r.	5%	33%
Muwanga 2008	100% (213)	17%	n.r.	26%	n.r.	n.r.	n.r.	57%
McGuire 2009	24% (49)	n.r.	n.r.	20%	n.r.	20%	10%	50%

n.r.; not reported.

### Causes of death in patients LTFU

The cause of death was investigated for 128 deaths in three studies from Johannesburg, South Africa [15], [16], [20], using verbal autopsy. For 81 patients (63%) the cause of death remained unknown. For the other 47 deaths, the reported cause of death was an AIDS defining illness in 29 patients (62%), a condition not related to AIDS in 16 patients (34%) and an unnatural cause in two patients (4%).

## Discussion

This systematic review and meta-analysis of studies that traced patients who were LTFU in ART programmes in resource-limited settings showed that the outcome of over a third of patients remained unknown. All studies except one were conducted in sub-Saharan Africa and no study was done in children. Among African adults who were LTFU after starting ART and successfully traced, the combined mortality was 46%. Mortality ranged from 12% to 87% across studies, and was inversely associated with the rate of LTFU in the programmes. Incorrect or missing telephone numbers and addresses were often the reason why patients could not be located. Transfer to another programme, financial constraints and improving or deteriorating health were common reasons for not returning to the clinic.

We performed a comprehensive search of the literature, including of abstracts presented at three major HIV/AIDS conferences, thus minimizing possible publication bias. We identified studies of over 6,000 patients who were LTFU in ART programmes in 10 low- or middle-income countries. Sites were heterogeneous and included both rural and urban locations. The approach used to trace patients varied and included telephone calls, home visits and social networks. Our findings should therefore be applicable to other ART programmes, particularly in sub-Saharan Africa.

Definitions of LTFU and the assessment of reasons for not returning to the clinic were not standardized across studies, which precluded formal meta-analysis of these data, and information on causes of death was limited. Other limitations include the lack of information, in most studies, on the time of death. The limited information that is available from some studies [16], [17], [19], [20] indicates that patients were lost in the first few months of ART, and died soon thereafter. Data from the ART in Lower Income Countries (ART-LINC) collaboration[5] and other treatment programmes, for example the Médecins Sans Frontières (MSF) programmes in Malawi [28], and South Africa [29], showed that loss to follow-up and death mostly occur in the first six months after ART initiation.

A high risk of death in the first few months after starting ART is characteristic of resource-limited settings where most patients start therapy late with advanced disease [5], [29], [30]. However, mortality in patients LTFU is substantially higher than the mortality commonly reported in the first year of ART based on routinely recorded deaths and, censoring of follow-up in patients LTFU [5], [29], [31]. ART programmes with high rates of LTFU and poor ascertainment of deaths may therefore seriously underestimate mortality. Furthermore, mortality among patients LTFU differs depending on the rate of LTFU of the treatment programme: mortality declined with increasing rates of LTFU. In programmes with high rates of LTFU those LTFU might thus include a sizeable group of low-risk patients who self-transferred to another programme, for example because of a more convenient location of the new clinic, to avoid stigma or due to work-related reasons. The results of dedicated studies tracing patients LTFU can be used to correct naïve estimates of mortality in a given programme [6], [14], [18]. In the absence of such studies, the data from this systematic review provide a sensible range of estimates of mortality in patients LTFU which can be used in sensitivity analyses to adjust overall mortality.

In most studies an important proportion of patients could not be located, and mortality of those whose vital status could be ascertained may not be representative of all patients LTFU. Contact information that is absent, incorrect or out-of-date could be related to the risk of death. For example, healthier individuals may be more mobile than sicker patients, and more likely to leave the catchment area of the clinic in search of work. Conversely, patients providing incorrect details may be part of a vulnerable group, with little social support and low adherence to ART. If results of tracing studies are likely to be affected by selection bias, correction of mortality is again best done in sensitivity analyses, using a range of plausible values. Clearly, the quality and completeness of patient's contact details should be improved and regularly updated during follow-up. Of note, a recent survey [32] of electronic medical record systems used in ART programmes in lower-income countries found that well managed databases might contribute to retaining patients in programmes.

An understanding of the reasons for not returning to care is important to the design of effective and cost-effective ART programmes. Outreach teams that routinely trace patients, combined with other measures, can substantially reduce LTFU [32], but such teams are costly, and the emphasis should be on the prevention of LTFU. Transfer to another programme was common among patients found to be alive. Strengthening of referral systems and regular exchange of information between clinics, together with patient education could increase the recording of transfers and ensure continuity of care. Unsurprisingly, financial constraints were another common reason for not returning to the clinic. Direct and indirect costs related to the provision of care have been identified as major obstacles to access to ART, acceptance of ART [33] and adherence to treatment [34], [35]. Mortality in programmes that charge user fees has been shown to be higher than in those offering free treatment [5]. Decentralisation of services, task shifting to lay care providers, longer drug refill periods for stable patients, as well as provision of transport vouchers for those in need are some of the strategies that could address this issue.

Other important reasons for LTFU were improvements in health, adverse effects and feeling too sick to come to the clinic or being hospitalised. Reports of stopping care as a result of perceived improved health reflect a poor understanding of the chronic nature of the disease and the need for continued, life-long ART. The experience or fear of toxicities has been found to be associated with poor adherence in previous studies [36], [37]. These issues need to be addressed through training of care givers and preparing patients for ART. Interventions that are aimed at the individual (rather than groups) and provided over longer time periods (>12 weeks) have been shown to be effective in improving adherence to ART [38].

Stigma and social problems were also repeatedly mentioned. Fear of disclosure, social isolation or the exposure to a discouraging social network have being identified as barriers to treatment adherence in studies conducted in high and low-income settings [35], [39]. In a study conducted in Botswana, Tanzania and Uganda, patients reported difficulties in taking their drugs when they were among employers, co-workers or friends to whom they had not disclosed their HIV status [34]. The development of practical medication management skills in open discussions with patients could be beneficial in this context [38].

In conclusion, a substantial minority of patients LTFU cannot be traced and among those traced on average 46% of patients have died. Transfer to another programme, financial constraints and improving or deteriorating health were common reasons for not returning to the clinic. These findings have important implications both for patient care and the monitoring and evaluation of ART programmes in resource-limited settings.

## Supporting Information

Appendix S1 Search strategies
(0.05 MB DOC)
