# Supplementary material for: Mortality of Patients Lost to Follow-Up in Antiretroviral Treatment Programmes in Resource-Limited Settings: Systematic Review and Meta-Analysis
Source: PLoS One. 2009 Jun 4;4(6):e5790. doi: 10.1371/journal.pone.0005790 (PMC2686174; doi:10.1371/journal.pone.0005790)
Supplement: Appendix S1 — Search strategies (0.05 MB DOC) [file pone.0005790.s001.doc]

**Appendix S1:** Search strategies

Details of search in EMBASE

'human immunodeficiency virus infection'/exp/mj NOT ('europe'/exp OR 'australia'/exp OR 'north america'/exp) AND ('lost to follow-up' OR 'loss to follow-up' OR ('losses' AND 'follow-up') OR 'late patients' OR 'dropout' OR 'drop-out') NOT 'clinical trial'/exp AND [humans]/lim AND [2000-2009]/py

Details of search in PubMED

("2000/01/01"[PDAT]: "2008/12/31"[PDAT]) AND ("humans"[MeSH Terms] AND ("HIV Infections/drug therapy"[Mesh] OR "HIV Infections/mortality"[Mesh]) NOT ("Europe"[Mesh] OR "Australia"[Mesh] OR "north america"[MeSH Terms]) AND ("lost to follow-up"[All Fields] OR "loss to follow-up"[All Fields] OR (losses[All Fields] AND follow-up[All Fields]) OR "late patients"[All Fields] OR "dropout"[All Fields] OR "drop-out"[All Fields])) NOT "Clinical Trial "[Publication Type:NoExp]

Details of search in LILACS and INDMED

The databases have a similar structure. Literature search was in advanced mode and included English, Portuguese, Spanish and French terms.

|  |  | **Search** | **In field** |
| --- | --- | --- | --- |
| 1 |  | HIV OR VIH | Words |
| 2 | AND | dropout$ OR abandon$ OR (lost AND to AND follow AND up) OR (perdu$ AND de AND vue) OR (perdido$ AND de AND vista) | Words |
| 3 | AND | (2000 OR 2001 OR 2002 OR 2003 OR 2004 OR 2005 OR 2006 OR 2007 OR 2008 OR 2009) | Country, year publication |
